# Zoonotic leishmaniasis in China: Current status and challenges to elimination

**DOI:** 10.1371/journal.pntd.0014344

**Published:** 2026-05-21

**Authors:** Thamali Manathunga, Vanessa Barrs, Qian Han, Fang Fang, Jairo Alfonso Mendoza-Roldan, Marcos Antonio Bezerra-Santos, Filipe Dantas-Torres, Domenico Otranto

**Affiliations:** 1 Department of Veterinary Clinical Sciences, Jockey Club College of Veterinary Medicine and Life Sciences, City University of Hong Kong, Kowloon Tong, Hong Kong, China; 2 Centre for for Animal Health and Welfare, Jockey Club College of Veterinary Medicine and Life Sciences, City University of Hong Kong, Kowloon Tong, Hong Kong, China; 3 Hainan International One Health Institute, Hainan University, Haikou, Hainan, China; 4 College of Animal Science and Technology, Guangxi University, Nanning, China; 5 Department of Veterinary Medicine, University of Bari, Bari, Italy; 6 Aggeu Magalhães Institute, Fundação Oswaldo Cruz (Fiocruz), Pernambuco, Brazil; Institute of Continuing Medical Education of Ioannina, GREECE

## Abstract

Leishmaniasis is expanding worldwide along with sand fly vectors. In China, particular challenges arise from its broad climatic and ecological gradients, the diversity of phlebotomine sand fly species, and numerous potential mammalian and reptilian hosts. Two ecological types of visceral leishmaniasis (VL) are recognized: anthroponotic VL (AVL) caused by *Leishmania donovani* and zoonotic VL (ZVL) caused by *Leishmania infantum*, which is subdivided into mountain-type (MT-ZVL) where dogs are the main reservoir, and desert-type (DT-ZVL) with uncertain reservoirs. Over the last decade, VL has re-emerged in central and northern regions of China, with increasing MT-ZVL notifications and evidence of canine infections in geographical areas where transmission had not previously been reported. This review synthesizes three decades of literature on canine leishmaniasis (CanL) and its vectors in China, focusing on domestic and wild reservoirs in the maintenance of zoonotic VL. Molecular studies reveal substantial genetic heterogeneity within the *L. donovani* complex and frequent discordance between single-locus markers (e.g., ITS1, *cytb*) and higher-resolution approaches (MLST/MLMT). *Leishmania* infection in dogs is most common in western foci, with high detection rates ranging from 24.8% to 77.2%, especially in Gansu and Sichuan Provinces, where subclinical infections predominate. Also, evidence of co-circulating unclassified *Leishmania* lineages related to *Sauroleishmania* complicates diagnosis and surveillance. Vector ecology, including the endophilic/exophilic behavior of *Phlebotomus chinensis* and the role of *P. sichuanensis* at high altitudes, overlaps with environmental change, dog management, and increasing stray dog populations, thereby amplifying transmission risk. Based on available data, dog culling is ineffective for long-term control of CanL due to poor diagnostics, rapid replacement of culled dogs, and ecological complexity. Conversely, vector control and bite prevention, using topical pyrethroid repellents (collars, spot-on pipettes) and possibly oral isoxazolines, should be implemented in dogs as control measures after appropriate field evaluation. Key gaps include insufficient canine surveillance and validation of diagnostic tools, as well as incomplete characterization of wildlife reservoirs. Strengthened One Health surveillance, integrating genomics, vector studies, and reservoir investigations, is essential to guide targeted control and predict future spread.

**Methods:**

The PubMed, Scopus, Google Scholar, and Web of Science databases were searched from 1950 to 2025 for peer-reviewed publications reporting the genetic diversity of *Leishmania* species, the epidemiology and prevalence of zoonotic VL and CanL, and trends in their spatial and temporal distribution in China for this narrative review. Three search strings were employed in these databases to; (1) identify reservoir host and sand fly vectors for leishmaniasis in China, (2) identify the epidemiology (risk factors, re-emergence, trends) and control and prevention of leishmaniasis in China, (3) identify the diversity of *Leishmania* species in China. The protocols for database searches are provided in the Supporting Information. The China National Knowledge Infrastructure database was additionally searched to obtain *Leishmania* prevalence data in animals. Data were extracted from articles published in English and Chinese. After removal of duplicates, abstracts of 865 articles were screened for relevance, from which 279 full-text articles were reviewed to extract relevant information. Articles were included for final review (*n* = 129) if they contained data on molecular and/or serological *Leishmania* infection-rate data in dogs, the genetic diversity of *Leishmania* species in China, computational trends in VL, reservoir hosts for *Leishmania* species and, sand fly vectors in China.

## 1. Introduction

Leishmaniases are neglected sand fly-borne diseases caused by parasitic protozoa of the genus *Leishmania* (Kinetoplastida: Trypanosomatidae). They are mostly zoonotic and cause severe disease, especially in tropical and subtropical areas [1–3]. However, leishmaniases are also expanding their geographic ranges in both Europe and the Americas, in parallel with their sand fly vectors [4–8]. In endemic areas, leishmaniases impose a major public health burden and are associated with low socioeconomic status and health [9]. It is estimated that at least one billion people are at risk of leishmaniasis worldwide, with approximately one million new cases recorded annually [10]. Due to the multiplicity of *Leishmania* species, reservoir hosts, and vectors worldwide, the epidemiology of leishmaniases is complex, and information about their distribution is scattered and underreported [7]. While there is abundant data on leishmaniases worldwide, information about the disease in China is limited. The vast topography of China is described as a ‘three-step west-east staircase’ commencing from the Qinghai-Tibet Plateau to Shanghai on the eastern coast [11]. China’s climate, ranging from tropical in the south to semi-arid and arid in the north, is suitable both for the development of sand fly vectors (Diptera: Psychodidae: Phlebotominae) and for a large number of animal species that are potential hosts of *Leishmania* spp. [12,13].

Two distinct ecological types of visceral leishmaniasis (VL) have been described in China, distinguished by the causative *Leishmania* species, geographical characteristics, sand fly vectors, and reservoir hosts [13]. Anthroponotic visceral leishmaniasis (AVL) is caused by *Leishmania donovani* and transmitted among humans by *Phlebotomus longiductus* in Xinjiang Uygur Autonomous Region, northwest China, and by *Phlebotomus chinensis* in eastern and central China [13–15]. The second type, zoonotic VL (ZVL), caused by *L. infantum,* is classified into two subtypes, mountain-type zoonotic VL (MT-ZVL) and desert-type zoonotic VL (DT-ZVL), based on their distribution and epidemiology [13,15].

MT-ZVL predominates in hilly regions in western China (Gansu, Sichuan, Shaanxi, Shanxi, Henan, and Hebei Provinces), where the domestic dog serves as the primary reservoir host, and *P. chinensis* is the main vector [13,15]. DT-ZVL is restricted to northwestern desert regions, including Xinjiang, northern Gansu, and western Inner Mongolia [13]. In the latter, animal reservoir hosts remain unknown, and transmission occurs primarily through *P. wui* and *P. alexandri* [13,15]. In addition, the *L. donovani* complex has been identified in human cases of cutaneous leishmaniasis (CL) in the Karamay region of Xinjiang [16], where species identification was disputed with different techniques [16–19].

**Fig 1:**
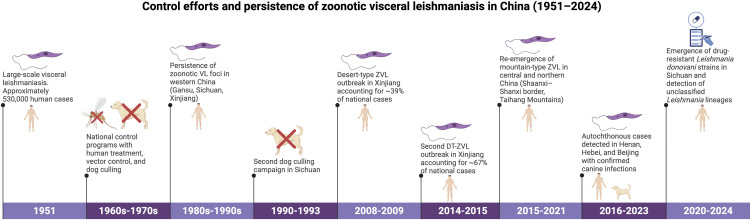
Timeline of key epidemiological events for leishmaniasis in China from 1951 to 2024. This figure illustrates major national control efforts, significant outbreaks, and re-emergence of zoonotic visceral leishmaniasis, and the emergence of *L. donovani*. Data synthesized from published literature. The figure was created in BioRender, Bezerra Santos, M. A. (2026) https://biorender.com/w3jg2sq.

Until the last decade, research on the distribution and epidemiology of both human and canine *Leishmania* infection in China was limited, and findings were often anecdotal, as original studies were published infrequently [20]. China was once considered highly endemic for VL, with 530,000 human cases reported in 1951 [14], and there are claims that it was eliminated by the 1970s [14]. This change in VL distribution was attributed to extensive control and prevention programs, such as those conducted in the plain regions of eastern and central China, which were based on treatment of half a million human patients and vector control [13,14]. However, transmission of ZVL never completely ceased, as the disease remains endemic in south- and northwestern China, with sporadic outbreaks. For instance, two outbreaks of DT-ZVL were recorded in Xinjiang during 2008–2009 and 2014–2015, accounting for 39% (393/1009) and 67% (494/798) of total human VL cases, respectively (Fig 1) [21]. Furthermore, recent surveillance data indicate that MT-ZVL remains present in central and western China, with a significant upward trend from 2015 to 2021 [22]. These data depict the complexity of evaluating the outcomes of elimination programs, given the main domestic and wildlife reservoir hosts relevant to the epidemiology of zoonotic VL. Indeed, apart from dogs, wild canids (e.g., red foxes, maned wolves, bush dogs), rodents, and lagomorphs have been identified as potential reservoirs for the sylvatic transmission cycle of *L. infantum* worldwide [23–26]. Therefore, the paucity of data on canine leishmaniasis (CanL) and on wildlife reservoirs limits understanding of disease dynamics in China, with data primarily restricted to southern and northwestern regions. This gap hinders a comprehensive understanding of the transmission cycle of zoonotic *Leishmania* infection in China.

In this review, we summarize the scientific literature on the epidemiology and biological aspects of zoonotic VL in China over the past three decades. We also discuss the roles of sand fly vectors and other domestic and wild animals that serve as reservoirs for *Leishmania,* as well as research gaps in the epidemiology of leishmaniasis in China.

## 2. Diversity of *Leishmania* species, their reservoirs, and vectors in China

**Fig 2:**
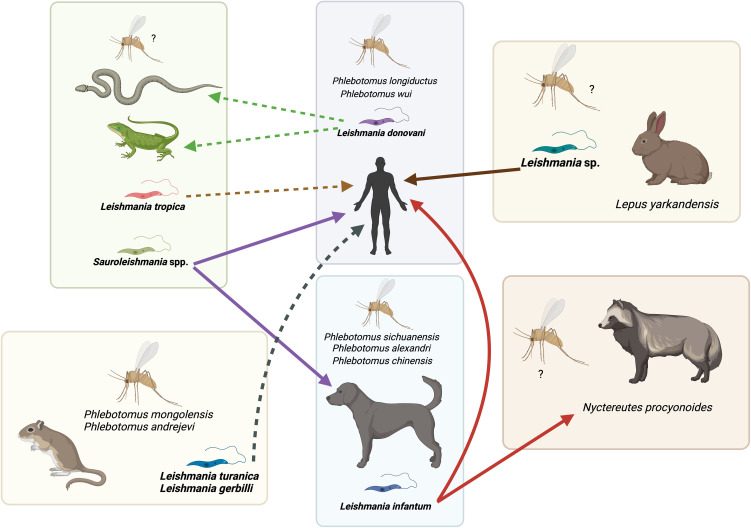
*Leishmania* species associated with their primary vertebrate host and Phlebotominae sand fly species in China, highlighting their interactions with other hosts. Colors of arrows represent different species of *Leishmania*: red, *Leishmania infantum*; light green, *Leishmania donovani*; dark green, *Leishmania turanica*, *Leishmania gerbilli*; light brown, *Leishmania tropica*; dark brown, *Leishmania* sp.; purple, *Sauroleishmania* spp. Solid arrows represent known associations and infections, whereas dashed arrows indicate limited information on infection and pathogenicity in China. Created in BioRender. Mendoza, J. (2026) https://BioRender.com/vkppnoz.

A wide variety of *Leishmania* species, such as *L. donovani*, *L. infantum*, *L. gerbilli*, *L. turanica*, *L. tropica*, and *Leishmania* (*Sauroleishmania*) spp. (Fig 2; Table 1) [17,27,28] have been reported in China with substantial genetic diversity and a complex evolutionary history [28].

**Table 1:** *Leishmania* species in China: associated hosts (reservoir and suspected), vectors, and geographic distribution.

*Leishmania* species	Vector	Host	Type of disease in humans	Geographic location	Ref.
*L. donovani*	*P. chinensis* (domestic)	Human*	AVL	Plain regions of Eastern and Central China	[13,14]
	*P. longiductus* (peridomestic)	Human*	AVL	Kashi, Xinjiang	[13,14]
*L. infantum*	*P. chinensis* (wild/peridomestic) *P. sichuanensis*	Dog*	MT-ZVL CanL	Hilly regions of Gansu, Sichuan, Shaanxi, and Shanxi	[29,30]
	*P. wui* *P. alexandri*	Unknown (Yarkand hare)	DT-ZVL	Xinjiang, northern Gansu, western Inner Mongolia	[13]
*L. turanica*	*P. mongolensis* *P. andrejevi*	Great gerbil*	_	Karamay, Xinjiang	[13,31,32]
*L. gerbilli*	*P. wui*	Great gerbil*	_	Karamay, Xinjiang	[13,31,32]
	*P. mongolensis* *P. andrejevi*	Great gerbil	_	Gansu and Inner Mongolia	[13]
*L. tropica*	Unknown	Lizards**	_	Desert region of northwest China	[33]
*Leishmania* (*Sauroleishmania*) sp. (close to *L. tarentolae*)	Unknown	Dogs**	_	Sichuan	[27,28,34–36]
		Lizard**	_	The desert region of northwest China	[37]

*Confirmed reservoir host.

**Detected only by molecular methods.

The *L. donovani* complex comprises *L. donovani* and *L. infantum*, which are the causative agents of AVL and ZVL in China, respectively [13]. Historically, these two forms of human VL were classified by the role of dogs in transmission, with *L. infantum* (the zoonotic type) in the northwest, where dogs are the major reservoir host, and *L. donovani* (the anthroponotic Indian type) in the east [37,38]. This involvement of two different species causing VL in China was further suggested by initial isoenzyme characterization of five human VL isolates, which were identified as *L. infantum* and *L. donovani* s.l. [39]. However, the geographic origin of these five human VL isolates does not align with the expected distribution. Two isolates from eastern China (Shandong Province), where the anthroponotic pattern predominates, were identified as *L. infantum*, and one isolate from Gansu Province in northwestern China, where zoonotic transmission was expected, was identified as *L. donovani* s.l. [39]. These findings should be interpreted with caution, as isoenzyme characterization limits the ability to discriminate among species.

Subsequently, DNA-based methods, including multilocus microsatellite typing (MLMT), fragment analysis, and multilocus sequence typing (MLST), have provided higher-resolution characterization of species diversity within the *L. donovani* complex [17,18,40]. Specifically, MLST analysis of five enzyme-coding genes (i.e., fumarate hydratase [*fh*], glucose-6-phosphate dehydrogenase [*g6pdh*], isocitrate dehydrogenase [*icd*], mannose phosphate isomerase [*mpi*], and 6-phosphogluconate dehydrogenase [*pgd*]) revealed that Chinese isolates of the *L. donovani* complex are genetically distinct from those isolates from India, Europe, and Africa [40]. These strains may have evolved independently within China over an extended period, rather than being introduced from other regions of the world. At the same time, MLST analysis demonstrated that strains with identical genotypes could be associated with different disease phenotypes (VL and CL), clustering by geographic origin rather than clinical presentation [40]. Thus, geographic location was a stronger determinant of genetic relatedness than disease manifestation [40].

Phylogenetic analysis of target genes is critical for determining species diversity, especially among closely related taxa. For instance, the cytochrome b (*cytb*) gene showed limited intraspecific variation among Chinese *L. donovani* isolates, compared to relatively greater variation in the internal transcribed spacer 1 (ITS1) region [27,35]. Nonetheless, even when the same marker was employed, different studies reached contrasting conclusions regarding species identification in China. For instance, two studies [35,41] used the ITS1 gene to analyze isolates from suspected VL patients in Jiashi county, Xinjiang. Whilst the first study identified the isolates as *L. infantum* [41], the second classified them as *L. donovani* [35]. Such discrepancies may reflect the phylogenetic complexity of *Leishmania* spp. in the region and highlight the need for more advanced molecular surveillance methods to support accurate diagnosis, assess epidemiological risk, and guide region-specific control measures.

To further complicate the already multifarious issues, a virulent strain of *L. donovani* (MHOM/CN/2016/SCHCZ) was isolated from the bone marrow of a human VL patient from Sichuan whose disease relapsed after antimonial treatment [42,43]. This strain exhibited greater virulence and antimonial resistance in promastigote culture, in infected macrophages, and in experimentally infected mice [42]. Furthermore, genomic analysis revealed high mutation rates in genes associated with antimony resistance (e.g., ABC transporter, ascorbate-dependent peroxidase, ATP-binding cassette protein subfamily A, among others) [43].

In addition, microsatellite multilocus typing of 29 *L. infantum* isolates from six endemic regions (Xinjiang, Gansu, Henan, Hebei, Shandong, and Sichuan Provinces) using 14 microsatellite markers revealed high genetic heterogeneity with 22 unique microsatellite profiles [17]. These Chinese strains formed two distinctive populations: one comprising 13 isolates that clustered with the *L. infantum* MON-1 group from Europe, the Middle East, Central Asia, and North Africa, and the other comprising 16 isolates that clustered with the *L. donovani/L. infantum* non-MON-1 group from Africa and the Indian subcontinent [17]. Interestingly, the Chinese strains in both groups formed unique clusters, clearly distinct from those in the two main clades above [17]. This pattern suggests long-term geographic isolation and independent evolution of these strains within China [17]. Accordingly, *L. infantum* from domestic dog blood samples from Beijing, northeast China, analyzed using MLST based on seven enzyme-coding genes (alanine aminotransferase [*alat*], enolase [*enol*], phosphoglucomutase [*pgm*], spermidine synthase [*spdsyn*], glucose-6-phosphate dehydrogenase [*g6pdh*], isocitrate dehydrogenase [*icd*], and mannose phosphate isomerase [*mpi*]), were also well separated from other global strains [44].

The causative species of leishmaniasis cases presented with cutaneous lesions is controversial. Indeed, molecular analyses, including MLMT and MLST, have shown that *Leishmania* spp. isolates from human CL and a local sand fly vector (*P. wui*) were genetically closer to *L. donovani* than to *L. infantum* [17,40]. Results from DNA hybridization studies [16,19] and inter-simple sequence repeat (ISSR) PCR [45] indicated that the same strains were more closely related to *L. infantum*. The *L. donovani* complex typically causes VL in the Old World. However, *L. infantum* has been associated with CL cases in Europe, whereas an atypical *L. donovani* strain has been identified as the causative agent of CL in Sri Lanka and parts of India [46–48]). These conflicting results highlight ongoing uncertainty in the molecular characterization of CL-causing *Leishmania* in Xinjiang, suggesting that further high-resolution genotyping is necessary to clarify the taxonomy and the molecular epidemiology of CL in China.

Furthermore, *L. major*, a species typically associated with CL in the Middle East and with the great gerbil (*Rhombomys opimus*) as the primary reservoir host in endemic regions, has been reported only occasionally among Chinese workers returning from Iraq [49], being imported from endemic areas of the Middle East [49,50]. Supporting this, *L. major* has never been detected in gerbil populations in China, *L. gerbilli* and *L. turanica* being the only species found in this animal host [31,32]. Although *L. turanica* is typically not associated with human disease, an isolate (MRHO/CN/88/KXG-2) from the ear tissue of a great gerbil in Karamay, Xinjiang, caused cutaneous lesions when experimentally inoculated into a human volunteer [31]. The isolate was identified as *L. turanica* by isoenzyme characterization [31] and phylogenetic analysis of various genetic markers (ITS1, *cytb*, *Leishmania* homolog of receptors for activated protein kinase C (*lack*)) [18,27,35]. The main vectors of *L. turanica* in China are *P. mongolensis* and *P. andrejevi* [31].

*Leishmania tropica*, the causative agent of anthroponotic cutaneous leishmaniasis (CL) in humans, has been rarely reported in China [51]. For example, an isolate from a human patient in Jiangsu Province, Eastern China, clustered with *L. tropica* in phylogenetic analyses of cytochrome oxidase II (*coxII*), *cytb*, and the small subunit ribosomal RNA (SSU rRNA) region [27,34,52]. Notably, *L. tropica* has been found in desert lizards (i.e., *Eremias vermiculata*, *E. velox roborowskii*, *E. multiocellata*, *Phrynocephalus axillaris*) in Xinjiang [33] though its role as a reservoir host for this infection remains unconfirmed [53].

Importantly, an undescribed *Leishmania* species, phylogenetically related to the subgenus *Sauroleishmania,* has been detected in both humans and dogs in China [27,28,34–36]. The species was isolated from asymptomatic dogs in Sichuan Province and identified using seven spliced leader (7SL) RNA segments, a highly conserved marker commonly used to differentiate *Leishmania* species [36,54]. Phylogenetic analysis showed that this undescribed *Leishmania* species formed a clade with *Leishmania* (*Sauroleishmania*) *tarentolae*, including a strain isolated from a human VL patient [36]. Most members of the subgenus *Sauroleishmania* are parasites of cold-blooded animals and non-pathogenic to mammals. There are some important exceptions of mammalian infection, such as *Leishmania adleri* in Africa that may cause human CL [55], and the non-pathogenic *L. tarentolae* molecularly detected in dogs, cats, and human blood in Italy [56–60]. This opens new opportunities for its use in vaccine development, given its ability to stimulate immune responses without causing disease [57,61]. Adding to the complexity of Chinese *Leishmania* isolates, some strains (MHOM/CN/54/#3, MHOM/CN/86/SC6, MCAN/CN/86/SC9) were initially identified as “undescribed” *Leishmania* sp. through *cytb* and *coxII* gene sequencing, but were later grouped with *L. infantum* based on MLST and MLMT analyses, revealing a lack of concordance between molecular markers and reinforcing the need for further taxonomic studies on *Leishmania* parasites in China [17,27,34,40]. More recent genome-wide analyses confirmed that some strains isolated from dogs and humans in China are indeed *L. infantum* [62]. An undescribed *Leishmania* sp. molecularly identified in asymptomatic dogs was phylogenetically close to a strain isolated from a symptomatic VL patient in Sichuan (MHOM/CN/90/SC10H2) [36]. Furthermore, several isolates obtained from human VL patients in mountain and desert foci were also identified as undescribed *Leishmania* sp. in phylogenetic analyses of ITS1, *coxII*, SSU rRNA, and 7SL RNA, suggesting a role in the epidemiology of human disease [34,35,52]. However, the transmission cycle, reservoir host, and zoonotic nature of this undescribed *Leishmania* sp. are yet to be defined. Further research may reveal that this undescribed *Leishmania* sp. represents an independent lineage within the *L. donovani* complex or that it is a hybrid, as in the case of *L. infantum* and *L. donovani* in Emilia Romagna, Italy, leading to a phenotypically more anthroponotic hybrid [63].

## 3. Canine leishmaniasis in China from the past to the present

Historical evidence of canine involvement in human VL transmission in China dates back to the 1950s. From 1951 to 1958, skin and/or bone marrow smears from approximately 120,000 dogs from endemic regions in northwestern and eastern China were examined [37]. Detection of amastigotes in these dogs showed that infected dogs were abundant in northwest (Gansu and Qinghai), north (Hebei), central (Henan), and northeast China (western highlands and peninsula in Liaoning Province), but rare in the eastern plains (Jiangsu, Anhui Province) (Table 2) [37]. This provided evidence that human leishmaniasis was highly associated with canine infection in the western and northern mountainous regions and, to a lesser extent, in the east [37]. Further supporting this pattern, blood-meal analysis of *P. chinensis* using the precipitin test showed that 12% of sand flies collected from Gansu (northwest China) had fed on dogs, whereas in Shandong Province (eastern China) it was only 1.8% [38]. This also underscores the importance of *P. chinensis*’ feeding preference in the distribution of canine infection. In addition, the above difference between eastern and western China was also observed in the age distribution of human cases: VL occurred mainly in infants and children in northwest China, whereas it was found in adults in eastern China [37]. These observations led to the early classification of two distinct epidemiological types of human VL in China: the Mediterranean type in the northwest, where dogs serve as the major reservoir host and are caused by *L. infantum*, and the Indian type in the east, where humans are considered the primary reservoir, which is caused by *L. donovani* [37]. While this classification was supported by early biological and basic epidemiological evidence, the findings should be interpreted with caution, given the limitations of diagnostic and species-identification methods available at the time. Furthermore, a considerable number of infected dogs were detected in northern Shandong Province (Table 2). However, the distribution of canine infection did not align with that of human VL, which was highly endemic in southern Shandong, where only 2 of 7618 dogs examined through skin/bone marrow smears tested positive [64].

**Table 2:** Human visceral leishmaniasis (VL) cases and canine leishmaniasis (CanL) cases from different provinces in China during 1951 to 1958 and CanL cases from 1959 to 1982 [37,65].

Province	Region	Human VL cases	CanL cases (1951–1958)			CanL cases (1959–1982)	Reference
			No. of dogs examined	No. of positive dogs	Positive dogs per 10,000	Positive dogs per 10,000	
Shandong	East China	5212	26,678	33	12.4	–	[37]
Jiangsu	East China	1,352	16,403	0	0	_	[37]
Anhui	East China	3,250	10,879	1	0.9	_	[37]
Henan	Central China	407	3,652	13	35.9	7.9	[37,65]
Hebei	North China	2,023	1,063	19	178.7	_	[37]
Shaanxi	Northwest China	3,414	41,037	156	38.0	38.1	[37,65]
Gansu	Northwest China	1,301	21,551	122	56.6	_	[37]
Hubei	Central China	15	571	0	0	_	[37]
Liaoning	Northeast China	_	539	11	204.1	183.1	[37,65]
Sichuan	Southwest China	228	1,320	2	15.2	388.5	[37,65]
Qinghai	Northwest China	57	687	9	131	–	[37]

Based on the above, dogs were considered reservoirs of *L. infantum* in the western mountainous region (northern Gansu and northwest Sichuan), thereby supporting a large-scale dog culling program regardless of dogs’ infection status [13,66]. However, the extent of dog culling during these critical years (1951–1958) was unknown, and its effectiveness is difficult to assess given that both canine infection and human VL cases continued to occur (Table 2) [64]. Later, a second dog-culling campaign was conducted in southwest China (Wenchuan and Lixian counties, Sichuan) between 1990 and 1993, with approximately 9,676 dogs euthanized and a transient reduction in human cases [67]. However, simultaneous insecticide spraying in areas where human cases occurred prevented a definitive conclusion about the effectiveness of culling programs. In addition, despite those efforts, both canine and human cases continued to increase and even spread to other non-endemic areas of Maoxian and Heshui counties in Sichuan Province [68,69].

**Fig 3:**
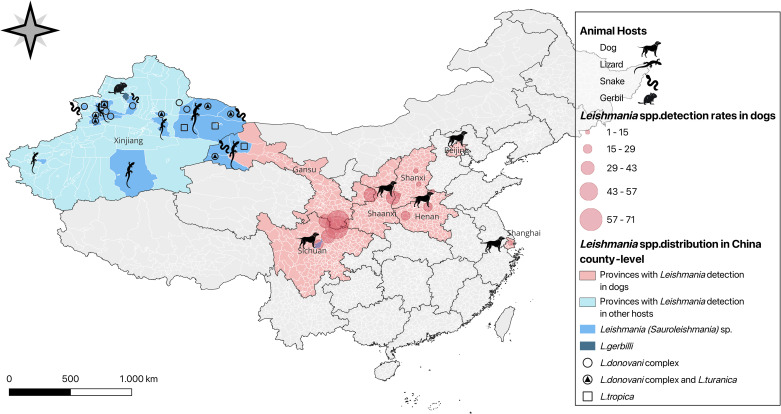
County-level distribution of *Leishmania* species, animal hosts, and canine leishmaniasis detection rates in China, 2005 to 2025. Animal silhouettes represent *Leishmania* spp. hosts detected in China. Red circles indicate the proportion of dogs testing positive via serological and/or molecular analysis at the county level. Light red fill shows provinces with *Leishmania*-positive dogs. Light blue fill shows the provinces with *Leishmania*-positive other animals. The base map uses province and county-level administrative boundaries from the geoBoundaries Global Database (https://www.geoboundaries.org/countryDownloads.html); license: CC BY 4.0 (https://www.geoboundaries.org/#tabs1-js). The animal silhouettes are from Free SVG, licensed under the CC0 public domain license (https://freesvg.org/). The map was created in QGIS version 3.44.9.

Despite all the control measures implemented in the western mountainous regions of China (i.e., culling of infected and uninfected dogs, prohibition of dog ownership, and deltamethrin bathing, alongside human patient treatment and vector control), CanL remains endemic (Fig 1) [13,70–72]. The molecular detection rate of *Leishmania* in dogs was high in Gansu (northwest) and Sichuan (southwest) provinces (Fig 3), ranging from 41.2% (221/537) [73] to 77.2% (61/79) [74] in Gansu and from 24.8% (78/314) [69] to 51.9% (55/106) in Sichuan, based on amplification of kinetoplast minicircle DNA (kDNA) (Table 3). The rate observed in these two provinces in western China is considered high compared with other endemic areas worldwide [75,76], further indicating that CanL remains prevalent in China. However, the kinetoplast minicircle network comprises different minicircle subclasses conserved across *Leishmania* species; therefore, most available PCR assays can amplify more than one minicircle subclass [77–79].

**Table 3:** Detection rates of canine leishmaniasis in China from 2005 to 2025.

Geographic region	City or County	Diagnostic methods	Sample type	Detection rate	Species identified	Ref.
Gansu	Wenxian, Diabu	cPCR (kDNA)	Blood	41.2% (221/537)	*Leishmania* spp.	[73]
Gansu	Wenxian	cPCR (kDNA)	Blood	77.2% (61/79)	*Leishmania* spp.	[74]
Gansu	Wudu	LAMP	Conjunctival swab	61.3% (68/111)	*L. infantum*	[80]
Sichuan	Wenchuan, Heishui, Jiuzhaigou	Real-time PCR	Blood	24.8% (78/314)	*L. infantum*	[69]
Sichuan	Jiuzhaigou	cPCR	Blood	51.9% (55/106)	*L. infantum*	[81]
Sichuan	Heishui	cPCR (kDNA)	Blood	28.6% (30/105)	*Leishmania* spp.	[82]
		ELISA	Blood	20.0% (21/105)	*Leishmania* spp.	[82]
Sichuan	Beichuan	cPCR (7 SL RNA)	Blood	22.0% (19/86)	*Leishmania* (*Sauroleishmania*) spp.	[36]
Shaanxi	Hancheng	rK39 ICT and ELISA	Serum	34.3% (115/335)	–	[83]
Shanxi	Yangquan	ELISA	Serum	6.0% (4/67)	–	[84]
Shanxi	Yangquan	rK39 ICT	Serum	7.4% (2996/40 573)	–	[85]
Shanxi	Yangquan, Wuxiang, Xiangning	rK39 ICT	Serum	6.4% (73/1149)	–	[86]
Henan	Zhengzhou, Luoyang, Anyang	rK39 ICT	Serum	18.8% (440/2342)	–	[87]
		cPCR (kDNA)	Blood	15.0% (139/929)	*L. infantum*	
Beijing	–	rK39 ICT	Serum	0.7% (30/4420)	–	[44]
	–	cPCR	Blood	0.9% (41/4420)	*L. infantum*	
Beijing	–	ELISA/qPCR	Serum/Blood	6.3% (36/575)	*L. infantum*	[88]
				(ELISA = 28, qPCR = 24)		
Shanghai	–	ELISA	Serum	5.9% (24/408)	*L. infantum*	[89]

Importantly, an unknown *Leishmania* species, closely related to *L.* (*S*.) *tarentolae,* was identified in 19/86 (22%) of canine blood samples in Sichuan Province (Beichuan county), and none of the dogs showed any clinical signs of CanL (Fig 3) [36]. Similarly, *L.* (*S*.) *tarentolae* has also been found in humans and dogs in southern Italy [60]. While *L. tarentolae* has been detected in lizards and snakes [90] in China (GenBank MH724806, MH724807, MK330211 - MK330214), these unknown *Leishmania* species in humans and dogs from the region cluster phylogenetically within *Sauroleishmania* yet being distinct from *L. tarentolae* sequences in phylogenetic analysis using *cytb*, ITS1-5.8S rRNA, and *hsp70* genes [35,91]. The pathogenicity of this unclassified *Leishmania* sp. in China remains unclear, and this finding raises the possibility that sylvatic *Leishmania* species may be circulating in the domestic dog population in Sichuan province. Additionally, the presence of multiple *Leishmania* species infecting dogs may contribute to the unusually high prevalence observed in western China.

As observed in other endemic regions [75,92,93], infected dogs with no apparent clinical signs are prevalent in China. For instance, molecular studies in Sichuan Province reported subclinical infection rates of 16.6% (52/314) [69] and 46.2% (49/106) [81]. Similarly, in Gansu Province, up to 40% (215/537) of dogs were reported to have no clinical signs despite being molecularly positive for *L. infantum* [73], although this study did not investigate clinico-pathological abnormalities.

Recently, new human VL hotspots have been identified in central and eastern China, particularly along the Shaanxi-Shanxi border and in eastern Shanxi Province, through a spatio-temporal cluster analysis of data from the National Notifiable Infectious Disease Reporting System [22,94]. The above is a passive reporting system for notifiable diseases in China that covers all health administrative authorities from the national level to local clinics [95]. The reports were classified as MT-ZVL, raising concerns about the occurrence of CanL in these areas [22,94], where seropositivity for *Leishmania* spp. was recorded in Shaanxi (34.3%) and Shanxi (5.9%) provinces (Table 3) [83,84,86]. Overall, because the serological assay was not sensitive enough to detect infection in asymptomatic dogs [92] and given the potential circulation of different *Leishmania* spp. in China, the actual prevalence of CanL could be considerably higher in these areas.

The number of human cases of MT-ZVL has also increased in the regions surrounding the Taihang Mountains (Henan, Hebei, and Beijing) in central China [96], an area previously considered endemic [87,94]. Indeed, 33 years after the last reported case (1984 in Henan), an autochthonous human VL case was reported in 2016 (Henan), followed by 16 cases in 2020, indicating an upward trend [87,97]. This resurgence prompted an investigation of the local canine population, revealing a relatively high prevalence of *L. infantum* in dogs (14.9%, 139/929) by molecular analysis (Table 3) [87]. In that study, ITS1 sequences from human VL patients and infected dogs showed more than 98% of homology, confirming a close epidemiological relationship between canine infections and human VL in the region [87].

Similarly, cases of VL caused by *L. infantum* have increased in Beijing (northern China), with the first case detected in 2019 [96]. The presence of *L. infantum* in Beijing has been historically documented through isolation from a wild raccoon dog (*Nyctereutes procyonoides*) [98]. However, epidemiological data on CanL in Beijing are scarce, with a positivity rate of 1.2% (54/4420) detected by both the rK39 immunochromatographic test (ICT) and PCR (ITS1) (Table 3) [44]. Notably, the positivity rate in dogs from the mountain areas of Beijing was higher than in dogs from the plains (45/961; 4.7% vs 9/3459; 0.26%), and five of the positive dogs from the plains had a history of travel to the mountainous region [44]. Furthermore, more than 30% of stray dogs in the mountain areas of Beijing were positive [44], though this was lower than in western regions of China (i.e., Gansu and Sichuan: up to 77.2% [74]. These findings may indirectly suggest that sand fly vectors prefer high-altitude habitats, underscoring the need for enhanced surveillance and control in these areas. A similar low seroprevalence of 5.9% (24/408) was reported in the household dog population in Shanghai (eastern China) [89], possibly reflecting improved care and management by dog owners.

## 4. Drivers of the re-emergence of leishmaniasis

Several environmental, socioeconomic, and terrain factors have been identified as key drivers of the re-emergence and spread of VL across Beijing, Hebei, Shanxi, and Henan Provinces between 2006 and 2023 [94,96]. Among these, long-term environmental changes (i.e., standardized precipitation/temperature deviation, forest cumulative change ratio, urban cumulative change ratio, crop cumulative change ratio) had the greatest impact, contributing 66.2% to overall risk, followed by socioeconomic (19.6%) and terrain-related (14.2%) factors [96]. Notably, VL cases were most strongly associated with increases in precipitation (~18%) and temperature (9.4%) [96]. Additionally, expansion of forest area (12.3%) and higher vegetation density (normalized difference vegetation index = 11.2%) were positively correlated with VL incidence, likely due to the creation of favorable habitats for sand fly vectors, such as exophilic *P. chinensis* [96]. In contrast, socioeconomic variables such as population size and GDP played a comparatively minor role [96].

In addition to changes in ecological factors, increased free-roaming dog populations and the introduction of *Leishmania*-infected dogs from endemic to non-endemic areas may also have contributed to the re-emergence [99]. Notably, stray and sheltered dogs serve as reservoirs for the zoonotic transmission of *L. infantum*, particularly due to their close contact, confined conditions in shelters, and the lack of preventive strategies [100]. In China, it was reported that the number of stray dogs exceeded 40 million in 2021, accounting for approximately 20% of the global stray dog population. Most dogs in rural areas of China are free-roaming and kept as guard dogs or companion animals [101].

Although studies on CanL in stray and sheltered dog populations in China are limited, research has emphasized the significant role of stray and free-roaming dogs in the re-emergence of *Leishmania* infection [44,87,102]. For instance, a retrospective case-control study in Shanxi Province, northern China, reported that the presence of stray dogs nearby increased the risk of *Leishmania* infection in humans 2.8-fold (OR = 2.767, 95% CI: 1.5–5.103, *P* < 0.01) [102]. Furthermore, dog breeding was a significant risk factor, with the presence of breeding dogs at home and in the neighborhood increasing the infection risk 4.2 [OR = 4.215, 95% CI: 2.027–8.763, *P* < 0.01] and 1.9 [OR = 1.953, 95% CI: 1.05–3.633, *P* = 0.035] times, respectively [102].

## 5. Transmission dynamics of CanL in China: From sand fly vectors to wildlife reservoirs

The transmission dynamics of *Leishmania* infection are complex, involving multiple interactions among the parasite, the reservoir host, and the sand fly vector [103]. Of the 47 sand fly species described in China, *P. chinensis* is the most widely distributed, occurring mainly across the northwest, north, northeast, southwest, and central parts of the country [12]. Notably, *P. chinensis* exhibits regional ecological variation, being endophilic (predominantly indoors) in the endemic plains of eastern China (i.e., Shandong, Jiangsu, Anhui, and Shaanxi Provinces), where AVL has been reported [14], and exophilic (predominantly outdoors) in endemic areas in western China, where CanL is predominant [14]. The variability in the distribution of CanL across China may be due to the behavior (exophilic versus endophilic) and/or feeding preferences of *P. chinensis*, but this remains poorly understood [64].

In high-altitude areas of Gansu, Sichuan, Yunnan, and the Tibet region, *P. sichuanensis* has also been identified as a vector of MT-ZVL in China [12,29,30,104]. Its vector competence was supported by the detection of *Leishmania* flagellates in the pharynx, anterior and posterior midgut, and hindgut [65].

Notably, *Leishmania*-infected (as indicated by the presence of promastigotes) *P. chinensis* was detected in the western mountainous region at altitudes of 1,500–2,200 m [105]. However, human and canine infections occurred only below 1,600 m, while areas above 2,000 m were uninhabited [105]. These findings suggest a natural nidus exists in high-altitude mountainous regions beyond human-canine transmission cycles [105]. Although direct evidence of wildlife reservoirs is sparse in China, repeated introduction of *Leishmania* infection into dog populations from sylvatic foci is one possible explanation for the failure of control measures, including mass dog culling.

Between 1951 and 1966, a total of 8,635 wild animals, including rodents, badgers, foxes, wolves, porcupines, cats, and hares, were examined, yet no amastigotes were detected in liver, spleen, lymph node and bone marrow smears [65,106], with the exception of a single raccoon dog captured in Beijing infected with *Leishmania* parasites [98], subsequently confirmed to be *L. infantum* [62].

Several molecular studies have been conducted in northwestern desert regions of China, particularly to resolve uncertainty about the reservoir host of DT-ZVL, where the dog population is relatively low [70]. Phylogenetic analysis of three genetic markers (heat shock protein 70 (*hsp70*), ITS1, and the gene encoding N-acetylglucosamine-1-phosphate transferase (*nagt*) showed that *Leishmania* isolates from Tarim hares, human VL patients from DT-ZVL foci in Xinjiang, and the sand fly vector (*P. wui*) clustered within the same clade, supporting the Tarim hare (*Lepus yarkandensis*) as a potential reservoir host in DT-ZVL [70]. Notably, both AVL and ZVL are present in Xinjiang, but in distinct geographic foci of endemicity: AVL in the southwest oases (Kashgar alluvial plain and the Aksu oasis) versus ZVL in the desert regions (mainly surrounding the Tarim and Hami basins) [13]. Phylogenetic analysis of the ITS1 region and *hsp70* gene revealed that isolates from DT-ZVL foci in Xinjiang were closely related to *L. donovani* from China, whereas isolates from AVL foci of Xinjiang were closely related to *L. infantum* from Mediterranean regions (France, Spain, and Italy), the Middle East (Iran), and China (Beijing, Xinjiang) [70]. Zoonotic VL caused by *L. donovani* has been reported previously [107], whereas no reports of AVL caused by *L. infantum* have been documented. Hence, more attention should be paid to clarifying the species and epidemiological type of VL, as misreporting may occur when different *Leishmania* species coexist within the same region.

The potential role of desert reptiles as reservoirs of zoonotic *Leishmania* spp. in the northwest Desert region has also been studied. In northwest China, where DT-ZVL (Xinjiang, Gansu) is endemic, *Leishmania* molecularly positive samples from the blood of desert lizard species were closely related to mammal-infecting *Leishmania* spp. Of these, 23 isolates were identified by ITS1 analysis as *L. tropica*, and one isolate belonged to the *L. donovani* complex [33]. Similarly, 145/316 (46%) lizards from the same region tested positive for the *L. donovani* complex by *cytb* and *hsp70* gene analysis [108].

Additionally, phylogenetic analysis of the ITS1 region revealed *Leishmania* (*Sauroleishmania*) spp. in blood samples from desert lizard species (*Eremias vermiculata*, *Eremias velox roborowskii*, *Eremias multiocellata*, *Phrynocephalus axillaris*, and *Tenuidactylus elongatus*) [33]. These sequences were closely related to *L. tarentolae* and *L. adleri*. Notably, isolates from lizards and dogs clustered within the same clade, indicating transmission of *Leishmania* between these two host species. These *Leishmania* (*Sauroleishmania*) spp. isolates were not classified as *L. tarentolae* or *L. adleri* because they showed high genetic divergence from these species, with 16 and 20 mutations separating the Chinese isolates from *L. tarentolae* or *L. adleri*, respectively [33].

Sympatric occurrence of multiple *Leishmania* species was observed in 42% (77/183) of lizards in the DT-ZVL foci in northwestern China, including *Leishmania* (*Sauroleishmania*) spp., the *L. donovani* complex, *L. tropica,* and *L. turanica* (Fig 3) [108]. The majority (57) of samples had co-infection with both *Leishmania* (*Sauroleishmania*) spp. and the *L. donovani* complex [108]. These findings in China and elsewhere [56,57,59] are stimulating further studies on the suitability of lizards as hosts for species of the *L. donovani* complex. For instance, experimental infection of a desert lizard species (*Phrynocephalus przewalskii*) with mammalian strains (*L. infantum* strain MHOM/CN/2016/SCHCZ and *L. donovani* strain MHOM/CN/80/801) showed that the lizard cleared the parasite, with a significant upsurge in Th1-type cytokines compared to mammals (BALB/c mice) [109].

In addition to lizards, the potential role of snakes (i.e., *Psammophis lineolatus*, *Gloydius halys*, *Natrix tessellata*) in maintaining zoonotic *Leishmania* in DT-ZVL-endemic foci has been investigated. Similarly, co-infection with multiple *Leishmania* species, including the *L. donovani* complex, *L. turanica*, and *L.* (*Sauroleishmania*) sp., has been detected in liver samples from desert snakes in the northwest desert regions of China (Xinjiang and Gansu) by analyzing three genetic targets: *cytb*, *hsp70*, and ITS1 (Fig 3) [90]. The molecularly identified reptilian *L.* (*Sauroleishmania*) spp. were closely related to strains from VL patients in China [90]. Taken together, these findings underscore the complexity of VL in China and highlight the need for further research to investigate the reservoirs of various *Leishmania* spp. in the country.

Although studies of wildlife reservoirs in the northwest desert region (DT-ZVL foci) have been conducted, apart from a few early reports, no recent studies have systematically investigated potential wildlife reservoirs in other regions of China. Given the re-emergence of MT-ZVL in several provinces and the expansion of wildlife conservation programs [110] there is a need to re-evaluate the role of wild mammals in *Leishmania* ecology. Understanding their contribution is vital for defining hidden transmission cycles and informing control strategies within a comprehensive One Health framework.

## 6. Risk factors associated with CanL in China

Host-related factors (e.g., age, sex, breed, and lifestyle) may influence individual variation in susceptibility to *Leishmania* infection [111,112]. Indeed, age has been reported as a significant risk factor for CanL in endemic regions, with older dogs showing higher infection rates, likely due to prolonged exposure to vector bites [69,89]. For example, in Sichuan Province, older dogs (>1 year) have a higher risk than younger dogs [69]. Moreover, a risk factor analysis in another CanL-endemic area (i.e., Gansu Province) found a higher risk in dogs younger than 2 years of age [73], consistent with the binomial distribution of infection in endemic dog populations, with peaks around 2–4 years of age and 7 years, respectively [73].

Although breed susceptibility is known to influence infection with *Leishmania* spp. [111,112], some studies in China have reported conflicting findings. For example, one study observed higher seroprevalence in crossbred dogs [89], whereas another reported an increased risk in purebred dogs [73]. Additionally, short-haired dogs were more susceptible to infection than long-haired dogs [73]. However, these data should be interpreted with caution, as many other factors, such as animal population background and environmental variables, should be considered. For example, dog lifestyle and management are critical factors for CanL [113], as dogs kept outdoors, particularly those tied outside, are at greater risk than those kept indoors [73]. Moreover, free-ranging dogs are at higher risk of *Leishmania* infection because they can access wild habitats where the vector *P. chinensis* predominates, thereby contributing to disease transmission and parasite maintenance in the area [114,115].

## 7. Diagnosis of CanL

Diagnostic tests with high sensitivity and specificity are critical for accurate identification of *Leishmania* spp. exposure in dogs. In China, the recombinant K39 (rK39) ICT is the most used serological assay [36,44,81,87], followed by enzyme-linked immunosorbent assays (ELISA) [36]. Although the rK39 ICT has shown higher sensitivity and specificity than IFAT in Europe [116], its performance may vary geographically [117]. In Brazil, for instance, the same test showed low sensitivity (59.3%, 95% CI: 37.9%–77.6%) despite high specificity (98.7%, 95% CI: 89.5%–99.9%) [118]. Such variation raises concerns about test accuracy in China as well, particularly given the presence of multiple *Leishmania* spp. infecting dogs. To date, few studies have assessed the diagnostic performance of rK39 immunochromatographic strips against molecular methods in Chinese canine populations. One of such study reported a low sensitivity of rK39 in dogs (65.2%) and humans (64.3%) compared to PCR [119]. An ICT test based on the circulating antigen of viscerotropic *Leishmania* species has been developed in China and demonstrated high sensitivity (95.8%) and specificity (98.7%) for human VL diagnosis [80]; however, this test has not yet been evaluated for the diagnosis of CanL. Furthermore, a double-antigen sandwich homogeneous chemiluminescent immunoassay has been developed with high sensitivity (100%) and specificity (95.1%) for detecting antibodies against *Leishmania* in dogs in China [120], but field studies are needed to further assess its performance. This represents a significant gap, especially given the high prevalence of CanL reported in endemic areas. In addition, regionally validated diagnostic methods are essential for effective surveillance and control strategies. Differences in the sensitivity and specificity of diagnostic methods may partly explain discrepancies reported in some studies. For example, in the same region (Jiuzhaigou County, Sichuan Province) in the same year (2011), two studies targeting the same marker (kDNA) reported different results; a lower prevalence (24.8%; 78/314) [69] of CanL was observed by real-time PCR, whereas a higher prevalence (51.88%; 55/106) [81] by conventional PCR (Table 3), even though the latter is less sensitive than real-time PCR. The discrepancy could be explained by the age of the sampled dogs, as Shang and colleagues [69] sampled a high proportion (220/314; 70%) of younger dogs (less than 1 year old) compared to older dogs, which are at greater risk of infection [111,112]. Hence, it is crucial to define the dog population and the diagnostic method to consistently assess and compare CanL prevalence data across heterogeneous regions, such as in China.

## 8. Control and prevention of CanL

Control measures implemented in China include: nationwide diagnosis and treatment of infected VL patients, residual insecticide spraying for vector control, and elimination of infected dogs (extensively reviewed elsewhere [13]). However, culling of infected dogs has proven ineffective as a long-term control measure for CanL due to a lack of highly sensitive and specific diagnostic tools to identify infected dogs and high replacement rates of eliminated dogs with susceptible puppies [20,121,122]. The limited impact of dog culling may also be partly attributed to ecological and epidemiological factors that influence the geographic distribution of CanL, such as vector density, vector feeding preference, dog lifestyle (e.g., outdoor vs indoor), environment (e.g., urban, suburban, or village), and the presence of wildlife and other reservoir hosts, including rodents and lagomorphs [1].

In addition to China, culling of infected dogs has also been implemented in highly endemic regions, such as Brazil, the Central Asian republics of the Soviet Union, and Palestine [123]. In Brazil, where both CanL and human VL are endemic, culling of infected dogs has failed to yield satisfactory results due to inconsistent and low annual screening coverage [122]. Additionally, in China, dogs were previously culled regardless of infection status, and in some regions, more than half were euthanized (~58% in Wenxian, Gansu province) [13].

Protecting animals from sand fly bites may have a longer-term effect than culling dogs to control zoonotic VL, but direct evidence from China is lacking. Evidence from the Transcaucasian and Central Asian Republics of the former Soviet Union, where ZVL was prevalent, shows that a sharp reduction in ZVL was mainly achieved by applying pesticide (DDT) to dwellings within 500 meters of microfoci of infection, rather than by culling dogs [124]. Application of DDT was also successful in eastern China, where AVL occurs, but to a lesser extent in the western mountainous region, where ZVL is present [14]. This may be due to the exophilic behavior of *P*. *chinensis*.

Given the limitations of dog culling, the use of repellents such as pyrethroids (e.g., deltamethrin, flumethrin, and permethrin) in dogs (e.g., impregnated collars or spot-on formulations) is the first-line approach to reduce the risk of phlebotomine sand fly bites and, consequently, the risk of *Leishmania* spp. transmission to other animals and humans [123,125]. Indeed, repellents induce killing and antifeeding effects, and late mortality of sand flies with varying levels of efficacy under laboratory and field conditions, typically lasting from weeks to up to one year [125]. The frequency of application of pyrethroid formulations to prevent *L. infantum* infection in dogs varies by geographic area and specific epidemiological scenarios, though they have been shown to be efficacious in reducing the incidence of infection in endemic areas of southern Europe, both in spot-on [126] and as collars [127].

Similarly, deltamethrin-impregnated dog collars reduced the seroconversion rate among both dogs and children in Iran [128]. Given their systemic insecticidal activity and proven efficacy in reducing populations of phlebotomine sand flies in specific foci, oral isoxazolines may complement pyrethroids as a control strategy to prevent human and canine leishmaniasis in endemic areas [125,129]. However, optimizing such a strategy requires further field studies to confirm its effectiveness (insecticide-susceptibility status) in sand fly control.

## 9. Future perspectives

Although the prevalence of human VL in China has been reported to have declined significantly since the 1950s, largely due to extensive national control programs [14], this conclusion should be interpreted with caution, and the epidemiological understanding of *Leishmania* infection remains incomplete. A major knowledge gap concerns the identity of wildlife reservoir hosts for *L. infantum*, particularly in mountainous and desert regions. Addressing this uncertainty is essential for sustained disease control and to prevent sporadic re-emergence. Furthermore, additional research is required to elucidate the pathogenicity, evolutionary history, and epidemiology of the newly identified *Leishmania* spp. found in both humans and dogs. Overall, the phylogeny and classification of *Leishmania* isolates in China remain incompletely resolved [27]. Under the above circumstances, updated phylogenetic frameworks are needed to accurately characterize *Leishmania* species in China and distinguish between *L. donovani* and *L. infantum*, thereby improving understanding of the epidemiology, clinical manifestations, and control strategies of the disease. This underscores the urgent need for refined molecular tools for the diagnosis and accurate characterization of *Leishmania* species in China. Data herein examined also suggest that single-gene markers lack sufficient discriminatory power to identify species within the *L. donovani* complex [18,33,34]. Therefore, genome-wide studies [58] on several *Leishmania* spp. isolates from China could provide valuable data on the causative agents of VL and CL in this country. In addition, surveillance for canine leishmaniasis in China has primarily been limited to the western and southwestern regions, not to the central, southern, and eastern regions, despite the widespread distribution of the competent vector *P. chinensis* in those areas. The creation of distributional maps of CanL would also prompt the implementation of control strategies based on individual use of repellent pyrethroids in dogs: a priority for reducing the risk of *Leishmania* spp. transmission to animals, including humans.

Finally, the emergence of drug-resistant strains in endemic foci for zoonotic VL, such as in Sichuan Province, underscores the need for continuous surveillance of both dog and human populations. Therefore, strengthened and geographically expanded surveillance, integrated with molecular characterization and vector ecology studies, is urgently needed to guide public health strategies and anticipate future transmission risks in the People’s Republic of China.
